# Reliability of birth weight recall by parent or guardian respondents in a study of healthy adolescents

**DOI:** 10.1186/s13104-018-3977-2

**Published:** 2018-12-10

**Authors:** Zeinab Kassem, Charlotte Burmeister, Dayna A. Johnson, Heather Dakki, Christine L. M. Joseph, Andrea E. Cassidy-Bushrow

**Affiliations:** 1 Department of Public Health Sciences, Henry Ford Hospital, One Ford Place, 5C, Detroit, MI 48202 USA; 2 Department of Epidemiology, Rollins School of Public Health, Emory University, 1518 Clifton Rd, NE, CNR Room 3025, Atlanta, GA USA

**Keywords:** Birth weight, Developmental origins of health and disease, Race, Reliability, Self-report

## Abstract

**Objective:**

Birth weight, which can be an indicator for risk of chronic diseases throughout the lifespan, is one of the most commonly used measures in the study of developmental origins of health and disease. There is limited information on the reliability of parent/guardian reported birth weight by race or by respondent type (i.e., mother, father, other caregiver).

**Results:**

Birth weight was reported by a respondent for 309 of the 333 (92.8%) study participants; of these, chart obtained birth weight was available for 236 (76.4%). There was good agreement between respondent report and chart obtained birth weight. Over half (N = 145, 61.4%) of respondents reported a birth weight within ± 100 g of what was in the chart; 60.9% of black participants (n = 81) and 62.1% of white participants (n = 64) fell within 100 g. Overall, mothers were 3.31 (95% CI 1.18, 9.33) times more likely than fathers to correctly recall the child’s birthweight within ± 100 g (p = 0.023). Respondent reported birth weight is a reliable alternative to chart obtained birth weight. Mothers were found to be most accurate in reporting birth weight of the child. Race/ethnicity was not significantly associated with reliability of birth weight reporting.

**Electronic supplementary material:**

The online version of this article (10.1186/s13104-018-3977-2) contains supplementary material, which is available to authorized users.

## Introduction

Increasing evidence supports that the development of many chronic diseases in adulthood originates in utero [1]. Black infants are more likely to be born low birth weight or experience preterm birth than their white counterparts and similarly suffer disproportionately from many adult chronic diseases that appear to have a developmental origin, including hypertension and heart disease [2–5]. Differences in the gestational experience of blacks compared to whites may lead to disparities in adult disease [2].

Birth weight remains one of the most commonly used measures in the study of the developmental origins of health and disease. Birth weight is a measure easily obtained via questionnaire and has been shown to be highly accurate and reliable in a number of studies reporting maternal recall of child birth weight [6–8], making it an attractive alternative to a more labor or cost-intensive approach such as medical chart review or linkage to state birth certificate data. Cultural differences influence people’s perception of health [9], with known racial differences in the perception of overall healthy weight [10, 11]. Black women may also be less likely to receive health information during prenatal care [12], which may affect their recall of birth weight from the time of delivery. There is limited information on race-specific reliability of parent/guardian reported birth weight in a contemporary group of healthy adolescents, with non-white mothers more likely to slightly overreport birth weight and white mothers more likely to slightly underreport birth weight [13]. In the United Kingdom, a study found that British/Irish white mothers reported birth weights accurately in higher numbers than other ethnic groups [6]. The findings of this study as well as others examining parental recall [6–8] are based on European cohorts where racial and ethnic distributions do not mirror those of the United States. In addition, studies either solely focus on maternal report [6, 13] or do not distinguish between paternal or maternal report [7, 8]. There is a gap in the literature regarding accuracy of birth weight report among different types of caregivers.

The purpose of the current study was to extend these previous findings by examining the accuracy of self-reported birth weight by parent/guardian respondent in a study of healthy adolescents (ages 14–17 years) and to determine if there were racial or parent/guardian status differences in the accuracy of self-report.

## Main text

### Methods

Potential participants were identified by accessing the administrative data warehouse of Henry Ford Health System (HFHS), which provides medical care to 20% of the metropolitan Detroit population [14]. The current analysis is a secondary data analysis of a cross-sectional study designed to examine adolescent health, and the recruitment process is described in detail elsewhere [15]. Briefly, adolescents aged 14–17 who had a well-child visit with a HFHS pediatrician were identified. Recruitment letters for the parent or guardian of 1837 eligible adolescents were sent to invite them to participate in the study. Those who agreed were recruited for a single study visit between November 2009 and June 2011, where trained interviewers met both the adolescent and their parent or guardian. A total of 335 adolescents completed a study visit. One adolescent < 14 years of age at the time of visit was excluded because of their age and 1 adolescent with a high weight (> 158.8 kg) that was discordant with the electronic medical record (EMR) was also excluded, as likely this was not the individual recruited in the study [15]. The final study sample consisted of 333 adolescents. Study protocols were approved by the Institutional Review Board at Henry Ford Health System (IRB #5410) and all adolescents provided written informed assent along with parental/guardian written informed consent. The Henry Ford Health System Institutional Review Board operates under a Federal Wide Assurance with the Department of Health and Human Services (#00005846).

#### Assessment of birth weight

During the interview, the parent/guardian was asked to self-report the child’s birth weight. Only children who were born at or who had their initial well-baby visit at HFHS-affiliated facilities had chart birth weight available. Using the child’s or mother’s unique medical record number, trained chart abstractors searched the EMR for each participant to obtain the weight at birth; paper charts were requested for those participants who did not have data in the EMR.

#### Covariate measurement

At the research clinic visit, adolescent’s height was measured with a wall stadiometer and weight with an electronic balance; body mass index (BMI) was calculated (kg/m^2^). Height, weight and BMI percentiles were calculated using the 2000 Centers for Disease Control and Prevention growth charts. BMI ≥ 85th percentile was considered overweight. Parent/guardian participants self-reported race/ethnicity, whether or not they were the biological parent of the adolescent, and the relationship type (i.e., mother, father, or other).

Participant primary residence address was obtained and geocoded to the 2000 US Census block. Residential education level was defined as the percent of households within a census tract with at least a high school education. Those residing in Detroit were considered to have an urban residence.

#### Statistical analysis

All analyses were conducted using SAS 9.4 (SAS Institute Inc, Cary, NC). Differences in participant characteristics were compared using independent *t* test for continuous variables and Chi square test of independence or Fisher’s exact test, if conditions were not met, for categorical variables. Bland–Altman plots were used to display differences between chart and respondent reported birth weights versus the mean of the chart and respondent reported birth weights. Univariate logistic regression models were used to model the association of participant demographics to whether or not the respondent was within 100 g of the child’s chart obtained birth weight. Spearman’s rank correlation coefficient was used to calculate correlation between actual and reported birth weight, broken down by patient demographics. The absolute value of the difference between chart and reported birth weight was compared by patient demographics with Wilcoxon signed rank test, since conditions of normality were not met.

### Results

Birth weight was reported for 309 of the 333 (92.8%) study participants (Additional file 1); of these, chart obtained birth weight was available for 236 (76.4%). Of those with respondent reported birth weight, there were no differences among the 236 with a chart obtained birth weight compared to the 73 without chart obtained birth weight, except mean BMI (p = 0.034) and proportion with a BMI ≥ 85th percentile (p = 0.018) was statistically significantly lower in those with a chart obtained birth weight (24.5 ± 6.3 kg/m^2^; 35.2%, respectively) compared to those without (26.3 ± 6.4 kg/m^2^; 50.7%, respectively).

We compared participant characteristics by availability of respondent reported birth weight (Table 1). Biological parents compared to others (p < 0.001) and mothers compared to fathers or others (p < 0.001) were more likely to have self-reported a child’s birth weight. Although not statistically significant, children with a respondent reported birth weight had a lower birth weight obtained from the chart (p = 0.079) compared to those without a respondent reported birth weight (3190.6 ± 689.4 g vs. 3490.6 ± 359.1 g, respectively). Race was not associated with respondent report of birth weight (p = 0.459).

**Table 1:** Comparison of characteristics between those with a respondent reported birth weight vs. those without a respondent reported birth weight

Adolescent characteristic	Respondent reported birth weight	No respondent reported birth weight	p-value
	N = 309	N = 24	
Age (years)	16.4 ± 1.0	16.4 ± 1.1	0.863
Male gender	142 (46.0%)	10 (41.7%)	0.685
Black	169 (54.7%)	15 (62.5%)	0.459
Urban	136 (44.2%)	6 (25.0%)	0.068
Residential education level	82.3 ± 12.0^a^	79.6 ± 6.6^b^	0.086
Body mass index (kg/m^2^)	25.0 ± 6.4	23.3 ± 5.1	0.247
Body mass index (≥ 85th percentile)	120 (38.8%)	7 (29.2%)	0.348
Respondent biological parent	291 (94.2%)	15 (65.2%)^c^	*<* 0.001*
Respondent type			
Mother	268 (86.7%)	11 (47.8%)	*<* 0.001*
Father	23 (7.4%)	4 (17.4%)	
Other	18 (5.8%)	8 (34.8%)	
Number with birth weight available in medical chart	236 (76.4%)	16 (66.7%)	0.286
Birth weight from medical chart (g)	3190.6 ± 689.4	3490.6 ± 359.1	0.079

* p-value for F-statistic

^a^N = 275 reporting residential education level information

^b^N = 17 reporting residential education level information

^c^N = 23 reporting biological parent information

**Fig. 1:**
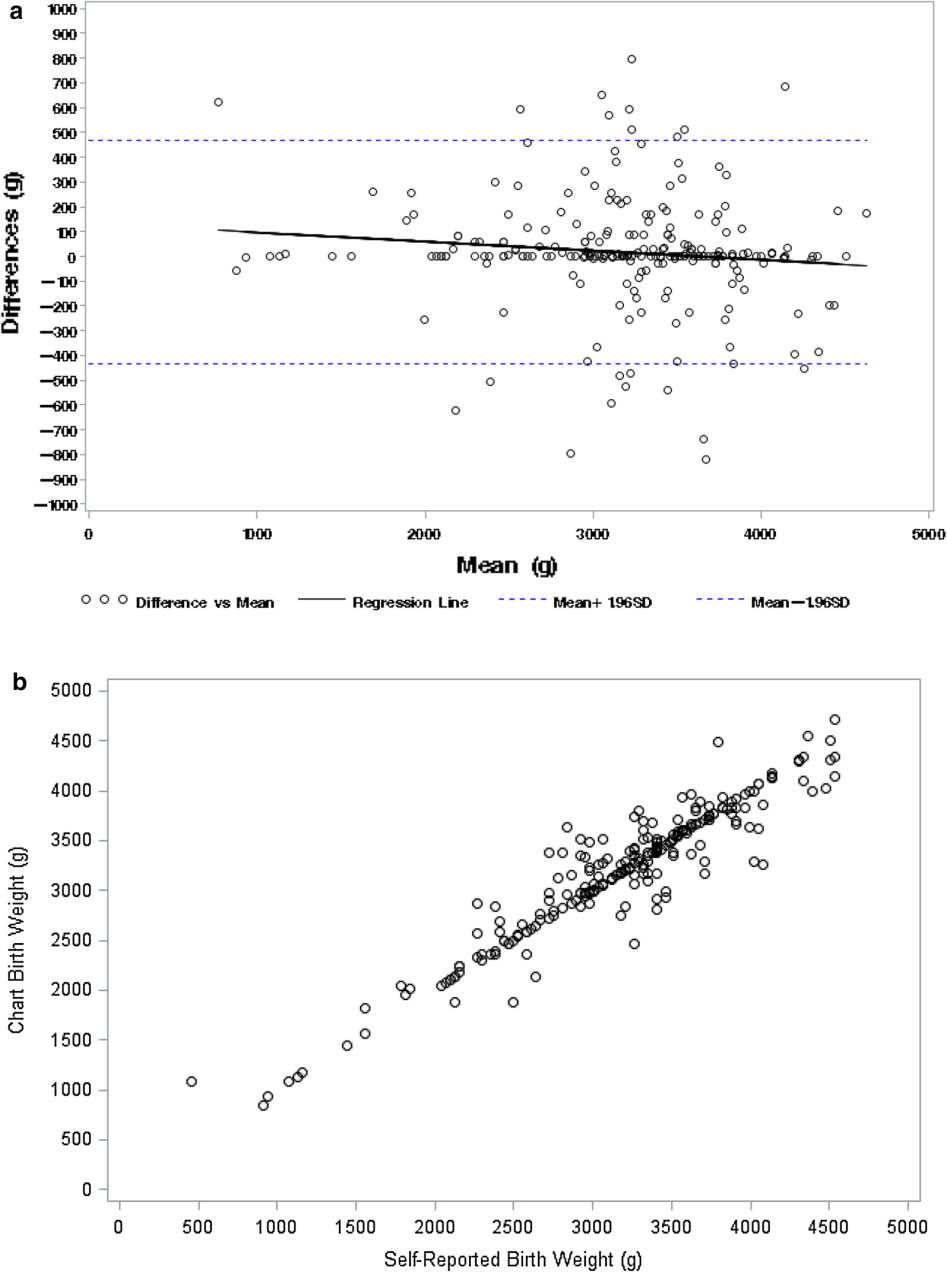
Graphs comparing respondent reported birth weight *vs* chart obtained birth weight. **a** Bland–Altman plot displaying differences between respondents reported birth weight and actual birth weight of the adolescents versus the mean of the respondents self-reported and actual birth weights of the adolescent; 95% limits of agreement are the dashed lines and the regression line is the solid black line. **b** Scatterplot of chart obtained birth weight compared to respondent reported birth weight

We examined potential discrepancies between respondent reported and chart obtained birth weight using Bland–Altman plots (Fig. 1a). In the overall sample, as well as stratified by race, participant results were similar across reporting methods, with respondent report and chart obtained birth weight having good agreement. The data points were evenly scattered around zero and across all birth weights, suggesting that there was no trend of disagreement. In addition, the scatterplot in Fig. 1b shows a strong positive linear relationship between chart obtained and respondent reported birth weight.

Both black (mean difference between respondent reported birth weight and chart birth weight = − 11.2 ± 237.3 g) and white participants (− 19.2 ± 222.8 g) tended to underreport birth weight, but the numbers were not significantly different (p = 0.792). Only 65 (27.5%) respondents self-reported a birth weight identical to the chart obtained birthweight; 33.1% of black respondents (n = 44) and 20.4% of white respondents (n = 21) (p = 0.031). We compared participant demographics between those who did and did not self-report birth weight within ± 100 g of the child’s actual birth weight (Additional file 2). Over half (N = 145, 61.4%) of all respondents reported a birth weight within ± 100 g of what was in the chart; 60.9% of black participants (n = 81) and 62.1% of white participants (n = 64) fell within 100 g. There was no difference by race (p = 0.847). Overall, mothers were 3.31 (95% CI 1.18, 9.33) times more likely than fathers to correctly recall the child’s birth weight within a ± 100 g margin (p = 0.023).

Finally we compared the absolute value of the median discrepancies of respondent reported birth weight and chart obtained birth weight across demographics (Table 2). The only significant difference was between respondent type (mother vs. father vs. other; p = 0.004). Mothers had a median absolute value of self-report vs. chart birth weight discrepancy of 28.3 g, fathers of 198.4 g, and others of 99.2 g. As previously, there were no differences based on race.

**Table 2:** Correlation and median discrepancy between respondent reported and chart obtained birth weight overall and by participant characteristics

Variable	r	N	Median discrepancy	p-value*
Overall	0.92	236	38.4	
Race				0.401
Black	0.92	133	35.4	
White or non-black	0.91	103	40.0	
Gender of child				0.260
Male	0.91	107	56.7	
Female	0.93	129	28.3	
Urban				0.254
Urban	0.91	109	21.8	
Not urban	0.92	127	56.7	
Body mass index percentile				0.993
≥ 85th percentile	0.90	83	28.3	
< 85th percentile	0.91	153	42.5	
Biological parent				0.166
Yes	0.92	222	34.5	
No	0.74	14	99.2	
Respondent type				0.004
Mother	0.93	205	28.3	
Father	0.86	17	198.4	
Other	0.74	14	99.2	

* p-value testing if median discrepancy is significantly different between the categories

### Discussion

Increasing evidence suggests an association between an individual’s in utero exposures and development of chronic non-communicable diseases later in life [1]. Low birth weight infants have higher rates of chronic illnesses throughout their lifetime [16]; therefore, it is important to have reliable and accurate birth weight measures to further examine these associations. Access to chart recorded birth weight is not always available, so researchers often rely on parent or guardian reported birth weight instead. In this study, we found high reliability of parent/guardian reported birth weight, suggesting that relying on the reported birth weight values is acceptable in research studies.

In a previous study, non-white mothers were more likely to slightly over-report birth weight (mean difference = 4.0 ± 16.6 g) while white mothers were more likely to slightly under-report birth weight (mean difference = − 8.3 ± 9.1 g) [13]. We specifically examined potential racial differences in the reporting of and the accuracy of reporting of birth weight as there are known racial differences in parental perception of healthy weight of children and in the receipt of health information during prenatal care [11, 12]. We found no differences by race, with both white and black respondents underreporting birth weight. Future studies that examine other racial/ethnic groups may be needed to ensure generalizability to the entire United States population which is racially and ethnically diverse.

In a previous study by Lucia et al. on a cohort of adolescents in Michigan, reliability of maternal birth weight recall within ± 250 g was 87.1% [13]. Our analysis resulted in similar findings when assessing reliability of maternal birth weight recall within ± 250 g (81.3% for black mothers and 81.7% for white mothers). A study by Tate et al., however, found that 92.4% of mothers reported birth weight within ± 100 g [6]. Although all three studies assess reliability of maternal birth weight recall, the pediatric cohort in Tate et al.’s paper is comprised of young children compared to adolescents in ours and Lucia et al., thus direct comparison is not possible. However, these three studies show that the reliability of respondent birth weight recall may also vary by the amount of time lapsed since birth of the child.

We identified several characteristics that were significantly associated with the reporting of birth weight or the accuracy of birth weight in our sample. Overall, biological parents were more likely to have respondent reported birth weight than other caregivers. In addition, mothers were more likely than fathers to correctly recall the child’s birth weight within a 100 g margin, suggesting maternal recall of birth weight is more accurate than paternal recall. Median discrepancies of respondent reported and chart obtained birth weight were also significantly different when comparing mother’s, father’s and other caregivers’ birth weight recall, with mothers having the smallest median discrepancy. Evidence has shown that women are more likely to participate in research than men [17]. Therefore, although fathers were less likely to accurately report birth weight, the contribution of this to a research study may be minimal. Additionally, the proportion of children being cared for by those other than their parents is considerable in the United States, particularly with respect to grandparents being the primary caregiver for grandchildren [18]. Birth weight recall by caregivers who are not the biological parents is overlooked. However, only 26 participants were neither mothers nor fathers, limiting our ability to make inferences and necessitating the need for additional studies to examine this aspect.

## Limitations

One of the main strengths of the study is the racial, educational and socioeconomic diversity of the study population as well as diversity in caregiver type (i.e., mothers, fathers or other). To our knowledge, the current study is one of very few conducted in the United States to include this type of diversity in caregivers. Another strength is the time interval between birth and collection of respondent reported birth weight during adolescence, confirming that reported birth weight continues to be a reliable measure well after birth.

A limitation of this study includes the relatively small sample size and small number of caregivers who were not the child’s mothers, which did not allow for the examination of reliability of respondent reported birth weight among this group. While we did not find any differences among participants who had available chart obtained birth weights compared to those who did not except for higher BMI among those without chart obtained birth weight, it is possible that our findings are subject to selection bias.

In conclusion, respondent reported birth weight is a reliable and efficient alternative to obtaining birth weight data from the medical record for use in research studies. In the current study sample, race was not associated with accuracy of respondent reported birthweight. Mothers were the most accurate in reporting birth weight of the child compared to fathers or other caregivers. Thus, caution should be exercised in using respondent reported birth weight when the person reporting the birth weight is not a biological parent, in particular, the mother. There were no other factors that were significantly associated with reliability of birth weight reporting.

## Additional files

**Additional file 1.** Study recruitment schematic. BMI, body mass index; DOHad, Developmental Origins of Health and Disease; EMR, electronic medical record.

**Additional file 2.** Univariable relationships of participant characteristics with whether or not the respondent was within 100 g of the child’s actual birth weight.

## Abbreviations

BMI: body mass index
EMR: electronic medical record
HFHS: Henry Ford Health System
