# Supplementary figures and images for: Reliability of birth weight recall by parent or guardian respondents in a study of healthy adolescents
Source: BMC Res Notes. 2018 Dec 10;11:878. doi: 10.1186/s13104-018-3977-2 (PMC6288863; doi:10.1186/s13104-018-3977-2)

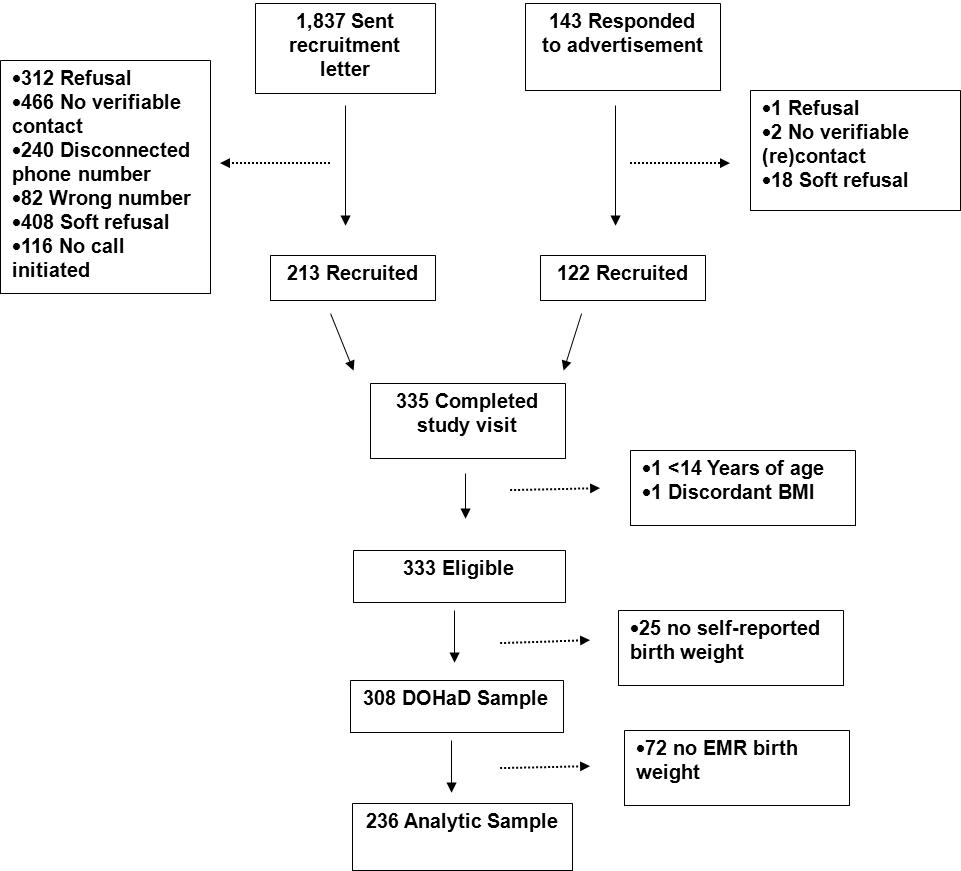

Supplement: Supplementary file 1 — Additional file 1. Study recruitment schematic. BMI, body mass index; DOHad, Developmental Origins of Health and Disease; EMR, electronic medical record. [file 13104_2018_3977_MOESM1_ESM.docx]
