# Supplementary material for: Reliability of birth weight recall by parent or guardian respondents in a study of healthy adolescents
Source: BMC Res Notes. 2018 Dec 10;11:878. doi: 10.1186/s13104-018-3977-2 (PMC6288863; doi:10.1186/s13104-018-3977-2)
Supplement: Supplementary file 2 — Additional file 2. Univariable relationships of participant characteristics with whether or not the respondent was within 100 g of the child’s actual birth weight. [file 13104_2018_3977_MOESM2_ESM.docx]

Additional file 2. Univariable relationships of participant characteristics with whether or not the respondent was within 100g of the child’s actual birth weight

| **Characteristic** | **Odds ratio (95% CI)** | **p-value** |
| --- | --- | --- |
| Male child | 0.661 (0.39, 1.12) | 0.124 |
| Age (years) | 0.922 (0.71, 1.20) | 0.544 |
| Black child | 0.949 (0.56, 1.61) | 0.847 |
| Residential education level | 0.990 (0.97, 1.01) | 0.405 |
| Body mass index | 0.998 (0.96, 1.04) | 0.907 |
| Body mass index (≥85^th^ percentile) | 0.856 (0.50, 1.48) | 0.576 |
| Urban | 1.002 (0.59, 1.70) | 0.994 |
| Biological parent | 1.643 (0.56, 4.85) | 0.369 |
| Respondent type |  |  |
| Other vs Mother | 0.553 (0.19, 1.64) | 0.285 |
| Mother vs Father | 3.31 (1.18, 9.33) | **0.023** |
